# Editorial: Application of ultrasound on peripheral neuromuscular disorders: From anatomy to clinic

**DOI:** 10.3389/fneur.2023.1138661

**Published:** 2023-01-23

**Authors:** Ke-Vin Chang, Alexander Grimm, Sang Beom Kim

**Affiliations:** ^1^Department of Physical Medicine and Rehabilitation, National Taiwan University Hospital, College of Medicine, National Taiwan University, Taipei, Taiwan; ^2^Department of Physical Medicine and Rehabilitation, National Taiwan University Hospital, Taipei, Taiwan; ^3^Center for Regional Anesthesia and Pain Medicine, Wang-Fang Hospital, Taipei Medical University, Taipei, Taiwan; ^4^Department of Neurology and Epileptology, Hertie Institute for Clinical Brain Research, Eberhard Karls University Tübingen, Tübingen, Germany; ^5^Department of Neurology, College of Medicine, Kyung Hee University Hospital at Gangdong, Kyung Hee University, Seoul, Republic of Korea

**Keywords:** ultrasound, sonography, nerve, muscle, pain

Ultrasound has been increasingly used in the evaluation of peripheral nerve diseases, such as carpal tunnel (1) and cubital tunnel syndromes (2), nerve tumors, and nerve traumas. The use of ultrasound has also been proven useful for the diagnosis of various kinds of hereditary and inflammatory neuropathies. With this in mind, a Research Topic focusing on the application of ultrasound on peripheral neuromuscular disorders has, thus, been initiated in the “Frontiers in Neurology” journal.

In this Research Topic, one narrative review and three original articles were included. Mezian et al. conducted a comprehensive review of ultrasound imaging and pertinent treatments for ulnar neuropathy. They incorporated several cadaveric pictures to elaborate on the course of the ulnar nerve and highlighted the structures along the cubital tunnel that commonly lead to entrapment. In addition to describing the existing protocol of ulnar nerve scanning, the authors also demonstrated the value of dynamic ultrasound for diagnosing ulnar nerve neuropathy. Besides emphasizing ultrasound imaging for diagnostic purposes, the techniques of ultrasound-guided hydro-dissection were also detailed.

Using dynamic ultrasound evaluation, Lo et al. investigated the change of median nerve mobility in patients with carpal tunnel syndrome before and after intervention (surgical decompression or corticosteroid injection). The authors employed a novel speckle-tracking method to quantify the average displacement of the median nerve during finger flexion and extension. Compared with the baseline, the nerve cross-sectional area decreased after the intervention, although no significant difference in the amplitude and curvature of the median nerve excursion was observed. The authors concluded that the nerve cross-sectional area would be more sensitive than its mobility for predicting the post-treatment outcome.

Siahann et al. investigated the thickness of piriformis muscles in participants with and in those without clinically diagnosed piriformis syndrome. The sonographic thickness measurement was standardized by evaluating the vertical distance between the superficial and deep fascia of the piriformis muscle at the medial edge of the ischium. They found an increase in piriformis muscle thickness in the patient group. Nevertheless, they did not measure the size of the underlying sciatic nerve, which might be associated with the symptom of sciatica.

Brünger et al. explored the role of ultrasound in differentiating patients with chronic inflammatory demyelinating polyneuropathy from those with non-inflammatory axonal polyneuropathies. The adjusted Bochum ultrasound score, ranging from 0 to 6, was thus, developed to summarize the sites of enlargement from the median, ulnar, radial, and sural nerves. There was a significant increase in the cross-sectional area of all the sampled nerves in the population with chronic inflammatory demyelinating polyneuropathy compared with that in participants with non-inflammatory axonal polyneuropathies. The inclusion of the adjusted Bochum ultrasound score improved the diagnostic capability, resulting in a sensitivity of 59% and a specificity of 94%.

In conclusion, ultrasound has been considered the most accessible resource to image peripheral nerves, and it can be readily used for clinical and research purposes. We are looking forward to seeing more advanced ultrasound techniques, such as sonoelastography (3, 4), to be applied to future investigations of various neuromuscular diseases.

## Author contributions

K-VC wrote the manuscript. AG co-edited the Research Topic. SK supervised the content of the draft. All authors contributed to the article and approved the submitted version.

## References

[1] Lin TY Chang KV Wu WT Ozcakar L. Ultrasonography for the diagnosis of carpal tunnel syndrome: an umbrella review. J Neurol. (2022) 269:4663–75. 10.1007/s00415-022-11201-z 35639198

[2] Chang KV Wu WT Han DS Ozcakar L. Ulnar nerve cross-sectional area for the diagnosis of cubital tunnel syndrome: A meta-analysis of ultrasonographic measurements. Arch Phys Med Rehabil. (2018) 99:743–57. 10.1016/j.apmr.2017.08.467 28888384

[3] Lin CP Chen IJ Chang KV Wu WT Ozcakar L. Utility of ultrasound elastography in evaluation of carpal tunnel syndrome: a systematic review and meta-analysis. Ultrasound Med Biol. (2019) 45:2855–65. 10.1016/j.ultrasmedbio.2019.07.409 31402226

[4] Hsu PC Chang KV Wu WT Wang JC Ozcakar L. Effects of ultrasound-guided peritendinous and intrabursal corticosteroid injections on shoulder tendon elasticity: A post hoc analysis of a randomized controlled trial. Arch Phys Med Rehabil. (2021) 102:905–13. 10.1016/j.apmr.2020.11.011 33338463

